# Draft genome sequence of endophyte *Bacillus atrophaeus* strain Adn01S isolated from *Agropyron desertorum* (Fish.ex Link) Schuit stem

**DOI:** 10.1128/mra.00844-25

**Published:** 2025-11-04

**Authors:** Vladimir K. Chebotar, Maria S. Gancheva, Margarita V. Khudyaeva, Oksana V. Keleinikova, Maria E. Baganova, Alexander N. Zaplatkin, Veronika N. Pishchik, Elena P. Chizhevskaya

**Affiliations:** 1 All- Russia Research Institute for Agricultural Microbiology, St. Petersburg, Russia; 2 Saint Petersburg State University, St. Petersburg, Russia; University of Strathclyde, Glasgow, United Kingdom

**Keywords:** draft genome, endophyte, *Bacillus atrophaeus*, Adn01S, *Agropyron desertorum*

## Abstract

We report on the genome sequence of *Bacillus atrophaeus* Adn01S, an endophyte isolated from the stems of *Agropyron desertorum* in Stavropol region, Russia. The genome was obtained using the Illumina NextSeq 1000 platform and consists of three scaffolds with a total length of 4 Mb and a guanine-cytosine content of 43.37%.

## ANNOUNCEMENT

Strain Adn01S was isolated from a surface-sterilized stem of *Agropyron desertorum* (Fish.ex Link) Schuit, a drought-resistant plant species, following the protocol described in reference 1. The samples were collected in arid regions of Stavropol Krai, Russia (44°44′31.0″ 45°01′47.0″E). The sterilization protocol included sequential washing with tap water (30 s), 70% ethanol (5 min), 15% H_2_O_2_ (10 min), and sterile water (five rinses, 2 min each). The tissue was homogenized under sterile conditions using a mortar and pestle. Aliquots (100 µL) of the resulting slurry were plated on 1/20-diluted tryptic soy agar (TSA; Difco Laboratories, MI, USA). Plates were incubated at 28°C for 3 days. A single colony of the strain Adn01S was inoculated into the plate with Luria-Bertani (LB) medium and grown at 30°C overnight. DNA was extracted from the grown bacterial culture using the Wizard Genomic DNA Purification Kit (Promega, USA). DNA library was prepared using the KAPA HyperPlus kit (Roche, Switzerland). The resulting library was sequenced using the NextSeq 1000 platform (Illumina, USA) that generated 1,304,166 read pairs (150 bp length).

The read quality was checked using FastQC v0.11.9 (2), thereby revealing high-quality data requiring no further processing. The draft genome was obtained with SPAdes v3.14.1 (3). The closest reference genome, *Bacillus atrophaeus* ASM3951917v1, was identified by FastANI v1.34 (4) (98.33% ANI) using reference genomes from the National Center for Biotechnology Information (NCBI) database (5). Substantially lower ANI values were observed with *Bacillus halotolerans* ASM400643v1 (82.40%) and *Bacillus mojavensis* ASM1264800v1 (82.27%). Scaffolding was performed with Ragtag v2.1.0 (6) using the reference genome ASM3951917v1. Contigs shorter than 500 bp were removed using the reformat tool from BBMap v39.08 (https://sourceforge.net/projects/bbmap/). The final assembly consisted of three scaffolds with a total length of 4,043,452 bp (ungapped length is 4,042,152 bp), a GC content of 43.37%, N_50_ of 4,039,118 bp, and average genome coverage of 96.7×. Reads were mapped to the assembly using bwa v0.7.18 (7), and genome coverage was calculated using samtools coverage tool v1.13 (8). Assembly statistics were evaluated using QUAST v5.1.0 (9). No plasmids were detected by PlasmidFinder-2.0 (10). The completeness of the genome was evaluated using BUSCO v5.2.2 (11) with the bacillales_odb10 lineage data set, and the BUSCO score was 99.8%. At the same time, CheckM v1.2.3 (12) indicated 99.12% completeness and 0% contamination. A total of 4,105 genes, including 3,873 coding genes and 75 RNA genes, were predicted on the assembly by NCBI Prokaryotic Genome Annotation Pipeline v6.8 (13). The biosynthetic gene clusters were identified using AntiSMASH v7.1.0 (14), with all extra features enabled. The analysis revealed clusters predicted to be associated with the synthesis of bacillibactin, subtilosin A, thailanstatin A, sporulation killing factor, surfactin, bacillaene, fengycin, and 1-carbapen-2-em-3-carboxylic acid. Unless otherwise noted, default parameters were used for all software. Previous studies have suggested that *Bacillus atrophaeus* may play a key role in enhancing plant stress resistance (15). Given its isolation from the tissues of a drought-resistant host plant (*Agropyron desertorum*), strain Adn01S represents a particularly promising candidate for further investigation of its stress-alleviating and plant growth-promoting potential.

## Data Availability

This project has been deposited at GenBank under accession number PRJNA1181152. The complete genome sequence was deposited under accession number GCA_044751405.1, and the raw reads in the Sequence Read Archive are under accession number SRR31201860.
